# Navigating the Controversies of Retromer-Mediated Endosomal Protein Sorting

**DOI:** 10.3389/fcell.2021.658741

**Published:** 2021-06-17

**Authors:** Yingfeng Tu, Matthew N. J. Seaman

**Affiliations:** ^1^Key Laboratory of Birth Defects and Related Diseases of Women and Children, Department of Pediatrics, West China Second University Hospital, Sichuan University, Chengdu, China; ^2^Cambridge Institute for Medical Research, University of Cambridge, Cambridge, United Kingdom

**Keywords:** retromer, sorting nexin, endosome, sorting, tubulation, controversies

## Abstract

The retromer complex was first identified more than 20 years ago through studies conducted in the yeast *Saccharomyces cerevisiae*. Data obtained using many different model systems have revealed that retromer is a key component of the endosomal protein sorting machinery being necessary for recognition of membrane “cargo” proteins and formation of tubular carriers that function as transport intermediates. Naturally, over the course of time and with literally hundreds of papers published on retromer, there have arisen disparities, conflicting observations and some controversies as to how retromer functions in endosomal protein sorting – the most note-worthy being associated with the two activities that define a vesicle coat: cargo selection and vesicle/tubule formation. In this review, we will attempt to chart a course through some of the more fundamental controversies to arrive at a clearer understanding of retromer.

## Retromer – The Early Observations

The analysis of specific yeast **v**acuolar **p**rotein **s**orting (*vps*) mutants led to the identification of the retromer complex (Seaman et al., 1997). The complex was determined to be a heteropentamer comprising five proteins; Vps5p, Vps17p, Vps26p, Vps29p, and Vps35p with one of each protein per complex (Seaman et al., 1998). Treatment with high salt buffers could dissociate the complex into two distinct subcomplexes; a dimer of Vps5p with Vps17p and a trimer of Vps26p, Vps29p, and Vps35p. The phenotypes of the different mutants, along with the localization of retromer proteins, clearly pointed to retromer functioning in the endosome-to-Golgi retrieval pathway. From biochemical data, it was postulated that the Vps35-Vps29-Vps26 trimer operated to select cargo proteins (e.g., the Vps10 protein that is a receptor for carboxypeptidase Y – CPY) whereas the Vps5-Vps17 dimer assembled to bend the membrane to make a vesicle. Thus, the two key properties of a membrane coat complex, namely cargo-selection and membrane-bending were encapsulated in the retromer complex.

This hypothesis was supported by some early observations but the initial data was far from conclusive. The proposed role of the Vps35-Vps29-Vps26 trimer in cargo-selection stemmed from the observation that Vps35p co-fractionated with Vps10p-containing membranes, even in a *vps29* mutant where Vps35p “followed” Vps10p to the vacuolar membrane fraction. No direct interaction between the cargo-selective complex and membrane proteins such as Vps10p was observed. The assembly of recombinant Vps5p into particles of a uniform size was taken as evidence that Vps5p, with Vps17p, would assemble *in vivo* to drive vesicle formation. But the direct evidence that Vps5p can drive membrane bending was not present when retromer was first reported.

## Data Supporting the Initial Hypotheses

Subsequent studies in yeast reported that Vps35p could be chemically cross-linked to a membrane protein that undergoes endosome-to-Golgi retrieval. Additionally, a mutation in the cytoplasmic tail of Vps10p rescued a mutation in Vps35p that, by itself, caused cargo-specific sorting defects (Nothwehr et al., 2000). These data strongly supported a role for Vps35p (and thus the Vps35-Vps29-Vps26 complex) in cargo selection. Furthermore, the use of the yeast two-hybrid system revealed an interaction between mammalian Vps35 and the cytoplasmic tail of the cation-independent mannose 6-phosphate receptor (CI-MPR), a membrane protein that functions analogously to the yeast Vps10 protein (Arighi et al., 2004). The use of RNAi to silence expression of Vps35 or Vps26 in mammals, along with experiments using cells derived from a Vps26−/− transgenic mouse, demonstrated that the trimer of Vps35-Vps26-Vps29 is necessary for the efficient endosome-to-Golgi retrieval of the CI-MPR and other cargo proteins such as sortilin, a membrane protein that is homologous to yeast Vps10p (Arighi et al., 2004; Seaman, 2004).

The Vps5 and Vps17 proteins belong to the sorting nexin (SNX) family that can bind phosphatidylinositol 3-phosphate (PtdIns 3-P), a lipid enriched on endosomal membranes (Horazdovsky et al., 1997; Burda et al., 2002). In higher eukaryotes, there are two Vps5p homologs, SNX1 and SNX2 that form heterodimers with either SNX5 or SNX6 – proteins that operate orthologously to Vps17p in yeast (Carlton et al., 2004; Wassmer et al., 2007). The carboxy-terminal halves of Vps5p or SNX1 or SNX2 comprise **B**in-**A**mphiphysin-**R**vs (BAR) domains that can assemble on liposome membranes and drive tubulation (Carlton et al., 2004; Peter et al., 2004). Hence, the dimer of SNX1/SNX2 with either SNX5 or SNX6 is often referred to as the SNX-BAR dimer. Live-cell microscopy of SNX1 tagged with green fluorescent protein (GFP) revealed that SNX1 is present on endosomal tubules *in vivo*. Thus a convergence of data showed that the sorting nexin element of retromer was capable of driving membrane bending although the carriers formed were not vesicles, but tubules instead.

## Sorting Motifs and Structures

The retrieval of the CI-MPR from endosomes to the Golgi requires a conserved three amino-acid motif in its cytoplasmic tail – tryptophan, leucine, methionine (WLM) (Seaman, 2007). A similar motif (FLV) in the tail of sortilin is necessary for its retrieval and a YLL motif in the cytoplasmic tail of the DMT1-II protein (an iron transporter) was also found to mediate its endosome-to-Golgi retrieval (Tabuchi et al., 2010). At the time these observations were published, it was assumed that recognition of these motifs would be through the trimer of Vps35-Vps29-Vps26 – frequently referred to as the cargo-selective trimer. The X-ray crystallographic structure of Vps29 showed it to be related to protein phosphatases and there is some data to support a role for Vps29 in dephosphorylating sites in the cytoplasmic tail of the CI-MPR (Collins et al., 2005; Damen et al., 2006). However, key residues necessary for phosphatase activity are absent from the putative active site of Vps29 and therefore the Vps29 protein may in fact play a purely scaffolding role in the Vps35-Vps29-Vps26 trimer (Swarbrick et al., 2011). Structural studies of mammalian Vps26 revealed its similarity to arrestin proteins that are known to select specific cargo for clathrin-mediated endocytosis (Hierro et al., 2007; Collins et al., 2008). Thus, the role of the Vps35-Vps29-Vps26 trimer in cargo selection was widely accepted.

## Two Complexes – Two Distinct Functions

Progress in understanding the function of retromer in mammalian cells led to the observation that the trimer of Vps35-Vps29-Vps26 does not readily associate with the SNX-BAR dimer (Harbour et al., 2010). Whereas, in yeast, the retromer proteins form a stable heteropentameric complex that associates with endosomes via the PtdIns 3P-binding properties of the Vps5-Vps17 dimer, in mammalian cells, the Vps35-Vps29-Vps26 dimer requires the small GTPase Rab7 for its recruitment to endosomes (Rojas et al., 2008; Seaman et al., 2009). In addition to Rab7, the SNX3 protein is also required for recruitment of the Vps35-Vps29-Vps26 trimer, an interaction that appears conserved in yeast where SNX3 is known as Grd19p and operates with retromer in endosome-to-Golgi retrieval (Strochlic et al., 2007; Harterink et al., 2011; Vardarajan et al., 2012). SNX3 (and Grd19p) does not have a BAR domain and therefore is quite different from the membrane-bending SNX-BAR dimer. Detailed high resolution microscopy of mammalian cells subsequently revealed that the Vps35-Vps29-Vps26 trimer and SNX dimer were detectable on distinct regions of endosomal membranes consistent with there being no stable association between the two complexes (Kvainickas et al., 2017a). The lack of any robust association between the Vps35-Vps29-Vps26 trimer and the SNX-BAR dimer in mammalian cells led to the term retromer being applied to just the trimer, sometimes with additional descriptors so that “core retromer” or “retromer cargo-selective complex” were used to describe the Vps35-Vps29-Vps26 trimer.

## New Data, a Change in Thinking

Possibly the most profound change in thinking with respect to how the retromer trimer and SNX dimer operate in endosome-to-Golgi retrieval occurred when it emerged that the SNX-BAR dimer associates with the WLM motif in the CI-MPR cytoplasmic tail and is necessary to sort the CI-MPR for endosome-to-Golgi retrieval (Kvainickas et al., 2017a; Simonetti et al., 2017; Yong et al., 2020). Previously it had been thought that the SNX-BAR dimer played a purely structural role – assembling into oligomers to drive tubule formation. The studies that identified the cargo-selecting role of the SNX-BAR dimer also reported that the retromer trimer of Vps35-Vps29-Vps26 was not required for the retrieval of the CI-MPR thereby up-ending nearly 20 years of established literature on retromer. This was quite surprising as many different studies, conducted independently in labs around the world, had observed the requirement for the retromer trimer in CI-MPR retrieval (Arighi et al., 2004; Seaman, 2004, 2007; Wassmer et al., 2007; Bulankina et al., 2009; Harbour et al., 2010; Hao et al., 2013; Follett et al., 2014; McGough et al., 2014b). Could they all be wrong?

The answer to that question is, probably not. Data obtained through an approach that causes cargo proteins to be “re-routed” to mitochondria revealed that there may be multiple carriers generated from endosomes that can transport proteins such as the CI-MPR to the Golgi (Cui et al., 2019). Some of these carriers depend on the function of the SNX-BAR dimer, others require the function of the retromer trimer. Thus, the spatial separation of the two complexes on endosomal membranes could provide the means by which the two sets of proteins function to sort proteins into distinct carriers. Cargo recognition by the retromer trimer may be mediated by Vps26, Vps35 or the SNX3 protein. Structural studies have revealed that SNX3, bound to the retromer trimer, can recognize the YLL motif present in the cytoplasmic tail of the DMTII-1 iron transporter (Lucas et al., 2016). The biochemical similarity between the WLM and YLL motifs also hints that SNX3 could sort the CI-MPR in a manner similar to the SNX-BAR dimer whilst the SNX-BAR dimer could function to retrieve proteins such as the DMTII-1 iron transporter. As SNX3 functions with the retromer trimer, this scenario would explain why multiple studies have reported that the Vps35-Vps29-Vps26 trimer, along with SNX3, is required for the endosome-to-Golgi retrieval of the CI-MPR.

## Arches That Drive Tubulation?

If SNX3, with the retromer trimer, can sort cargo proteins independently of the SNX-BAR dimer, how do these proteins form a vesicle or tubule without the self-assembly and membrane-bending activities of the SNX-BAR dimer? This question presently does not have a clear answer but clues have emerged recently from cryo-electron microscopy and tomographic studies that have imaged the retromer trimer on liposomes (Kovtun et al., 2018). It was observed that two copies of the Vps35-Vps29-Vps26 trimer can create rigid “arches” on artificial membranes. The arches decorated tubules formed from the liposomes. Although the study utilized a fungal version of the retromer complex where tubulation of the membranes could have been driven, at least in part, by the presence of a Vps5p homolog, it is tempting to speculate that the Vps35-Vps29-Vps26 trimer could contribute to the formation of the membrane tubules and might do so in mammals when in association with SNX3 and Rab7. If this is true, then the two distinct complexes; the SNX-BAR dimer and the retromer trimer (with SNX3 and Rab7) could both possess the two key activities required for sorting of proteins into an endosome-to-Golgi pathway; namely recognition of cargo and membrane bending. The two distinct complexes could preferentially sort different cargoes although it should be remembered that the SNX-BAR dimer and SNX3 protein can both bind motifs that are biochemically similar, the WLM and YLL motifs, respectively. Alternatively, the two complexes could mediate retrieval pathways that are kinetically distinct. That said, it remains to be determined if the propensity to form tubules *in vitro* translates to tubule-forming activity *in vivo* where the cytoplasmic milieu would possibly limit the extent to which a tubule could extend outward from an endosome. Additionally, not all studies of the retromer trimer have supported a role in forming tubules so some doubt remains as to important the retromer trimer is for tubule formation (Deatherage et al., 2020; Kendall et al., 2020).

## Connections to the Cytoskeleton

In mammals, the SNX-BAR dimer associates with the dynein motor through the p150glued/dynactin protein (Hong et al., 2009; Wassmer et al., 2009). This interaction links SNX-BAR dimer-mediated sorting with transport along microtubules toward the microtubule organizing center (MTOC) which is, in most circumstances, localized close to the Golgi complex. Presently, it is not known if the retromer trimer also has the ability to link with the microtubule cytoskeleton but it seems unlikely that it does as several studies using mass spectrometry to identify binding partners have not revealed any interaction between the retromer trimer and motor proteins such as Dynein (Harbour et al., 2010; McGough et al., 2014a). Thus, there remains a potential gap between the sorting and putative tubulating activity of the retromer trimer and a clear role in directing cargo proteins toward the MTOC and the Golgi complex like the SNX-BAR dimer.

The Vps35-Vps29-Vps26 trimer does, however, play an important role in the formation of filamentous (F)-actin at endosomes being necessary for the recruitment of the WASH complex to endosomal membranes. A direct interaction between the Vps35 protein and the extended unstructured “tail” domain of the Fam21 subunit (also known as WASHC2) of the WASH complex ensures that the WASH complex is endosomally localized at sites where the retromer trimer is presumably sorting membrane proteins (Harbour et al., 2010, 2012; Jia et al., 2012; Helfer et al., 2013). The role and organization of F-actin at endosomes has yet to be fully elucidated. However, data strongly supports the view that the retromer trimer, through its association with the WASH complex, directly contributes to recycling of membrane proteins from endosomes to the cell surface. Several membrane proteins including the transferrin receptor, the β2-adrenergic receptor and the Glut-1 glucose transporter require the retromer trimer and WASH complex for their endosome-to-cell surface recycling (Derivery et al., 2009; Temkin et al., 2011; Steinberg et al., 2013). Sorting of membrane proteins for this pathway requires SNX27, a non-BAR domain-containing SNX protein that binds to both Vps26 and the “tail” of Fam21. There is some conflicting data regarding the role of the WASH complex in the endosome-to-Golgi retrieval of the CI-MPR and it may be that loss of WASH complex function has somewhat wide-ranging effects on the endolysosomal system resulting in differences observed between various cell lines (Gomez and Billadeau, 2009; Harbour et al., 2010; Zavodszky et al., 2014). Some notable cargo proteins, e.g., the copper transporter ATP7a accumulate in endosomes after loss of WASH complex function although it has yet to be determined if this is due to reduced endosome-to-Golgi retrieval, or impaired endosome-to-cell surface recycling (Phillips-Krawczak et al., 2015). There is no WASH complex in yeast so its function is dispensable in a simple eukaryote (Derivery and Gautreau, 2010). Loss of WASH complex function is often accompanied by increased endosomal tubulation suggesting that part of the WASH complex function may to regulate the processes that govern tubulation, be it through SNX-BAR proteins or the hypothetical tubulating activity of the Vps35-Vps29-Vps26 trimer.

## Controversies Resolved?

Where does this leave the current understanding of the retromer complex and its role in endosomal protein sorting? In a simple eukaryote such as yeast, retromer is the heteropentameric complex that is required for the endosome-to-Golgi retrieval of membrane proteins such as Vps10p (Seaman et al., 1997, 1998). The process of cargo-selection requires the Vps35-Vps29-Vps26 trimer. There is substantial data to support a role in cargo-binding for both Vps26 and Vps35 but in addition, the Grd19 protein (homologous to SNX3) is likely to also sort specific cargo when associated with retromer (Nothwehr et al., 2000; Strochlic et al., 2007; Suzuki et al., 2019). The Vps5-Vps17 dimer may drive tubulation through self-assembly but this may be aided by the formation of “arches” by the Vps35-Vps29-Vps26 trimer. It is also possible that Vps5-Vps17 could directly sort cargo in a manner similar to the SNX-BAR dimer in mammalian cells.

**FIGURE 1:**
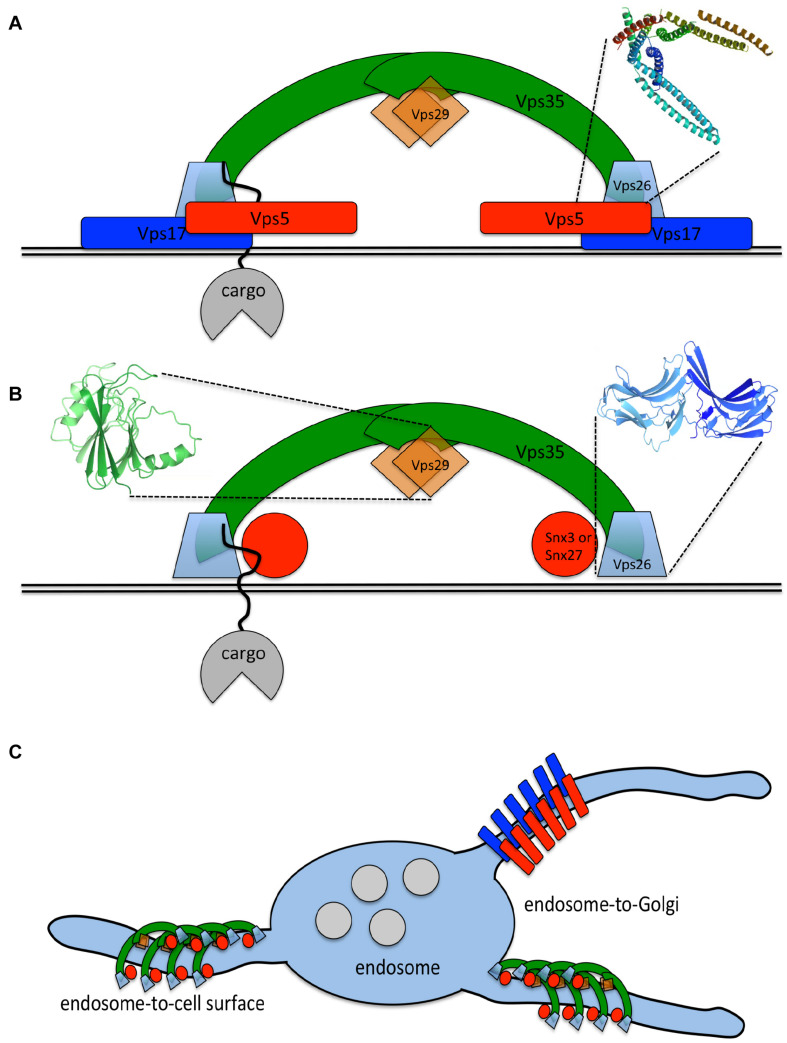
The retromer complex in simple or higher eukaryotes. **(A)** In a simple eukaryote such as yeast, the retromer complex is a stable heteropentamer that associates with endosomal membranes to sort cargo proteins (e.g., Vps10p) into tubules for endosome-to-Golgi retrieval. The Vps26 protein (along with Vps35p) plays a key role in cargo recognition but Grd19p (SNX3) also contributes to sorting specific cargo. The BAR domain (PDB:4FZS) of SNX-BAR proteins such as Vps5p and Vps17p adopts a curved conformation and can tubulate membranes. **(B)** In mammals, there is no stable association between the retromer trimer of Vps35-Vps29-Vps26 and the SNX-BAR dimer of SNX1/2-SNX5/6. Both sets of proteins can recognize and sort cargo with similar aromatic motifs being recognized by the retromer trimer (aided by SNX3 and SNX27) and the SNX-BAR dimer. The arrestin-like conformation of VPS26 (in blue, PDB:2R51) may be important in cargo-recognition. Both sets of proteins may also be able to tubulate the membrane, the SNX-BAR dimer via the tubulating activity of the BAR domain and the retromer dimer due to its arch-forming properties that requires VPS35 to fold around the globular VPS29 protein (structure shown in green, PDB:1Z2X). **(C)** The propensity to tubulate the membrane could enable the retromer trimer and SNX-BAR dimer to generate multiple distinct transport intermediates for trafficking from endosomes to both the Golgi and the cell surface. The destination of the different transport intermediates may depend on the sorting of correct SNAREs into a tubule or association with motor proteins that mediate transport via the microtubule cytoskeleton.

In more complex eukaryotes, the term retromer now describes the Vps35-Vps29-Vps26 trimer. This functions to sort cargo proteins, either through the activity of Vps26 or by association with SNX3 or SNX27 for endosome-to-Golgi retrieval or endosome-to-cell surface recycling, respectively (Harterink et al., 2011; Temkin et al., 2011; Fjorback et al., 2012; Steinberg et al., 2013). The different destinations of SNX3-retromer or SNX27-retromer cargo may by achieved through sorting of SNARE proteins required for fusion of the transport intermediate with its target membrane or through interactions with motor proteins. These aspects of retromer-mediated protein sorting remain to be elucidated. The SNX-BAR dimer can also sort cargo by recognition of motifs such as WLM in the cytoplasmic tail of the CI-MPR. There is a host of accessory factors that facilitate and augment sorting by the retromer trimer and/or SNX-BAR dimer including the WASH complex (Harbour et al., 2010; Kvainickas et al., 2017b). It is still widely believed that the SNX-BAR dimer drives tubule formation but the “arches” formed by the retromer trimer and the data indicating that multiple carriers operate in endosome-to-Golgi retrieval suggest that tubule formation may not be solely mediated by the SNX-BAR dimer (see Figure 1; Kovtun et al., 2018; Cui et al., 2019).

Thus, although tremendous progress has been made in deciphering the mechanisms by which retromer and the SNX-BAR dimer operate in endosomal protein sorting, some basic truths remain unchanged from the first reports on retromer more than 20 years ago: namely that the constituent proteins possess both cargo-selection and membrane bending properties necessary to sort cargo proteins into a transport intermediate. It is perhaps paradoxical that the greater clarity in understanding how retromer functions has led to a blurring of the once clear dividing line between the two activities of cargo recognition and membrane bending and the respective roles of the retromer trimer and SNX-BAR dimer.

## Author Contributions

Both authors listed have made a substantial, direct and intellectual contribution to the work, and approved it for publication.

## Conflict of Interest

The authors declare that the research was conducted in the absence of any commercial or financial relationships that could be construed as a potential conflict of interest.
